# Supplementary material for: Hyaluronic Acid as Macromolecular Crowder in Equine Adipose-Derived Stem Cell Cultures
Source: Cells. 2021 Apr 9;10(4):859. doi: 10.3390/cells10040859 (PMC8070604; doi:10.3390/cells10040859)
Supplement: Supplementary file 1 [file cells-10-00859-s001.pdf]

Table S1. Qualitative HA solubility assessment as a function of molecular weight and concertation. ✓ indicates soluble.

	**0.1 mg/mL**	**0.5 mg/mL**	**1.0 mg/mL**	**5.0 mg/mL**	**10 mg/mL**	**20 mg/mL**	**30 mg/mL**	**50 mg/mL**	**60 mg/mL**	**100 mg/mL**	**200 mg/mL**	**500 mg/mL**
10 kDa	✓	✓	✓	✓	✓	✓	✓	✓	✓	✓	✓	Too viscus / Gel
60 kDa	✓	✓	✓	✓	✓	✓	✓	✓	✓	Too viscus / Gel		
100 kDa	✓	✓	✓	✓	✓	✓	✓	Too viscus / Gel				
500 kDa	✓	✓	✓	✓	✓	Too viscus / Gel						
1,000 kDa	✓	✓	✓	✓	Too viscus / Gel							

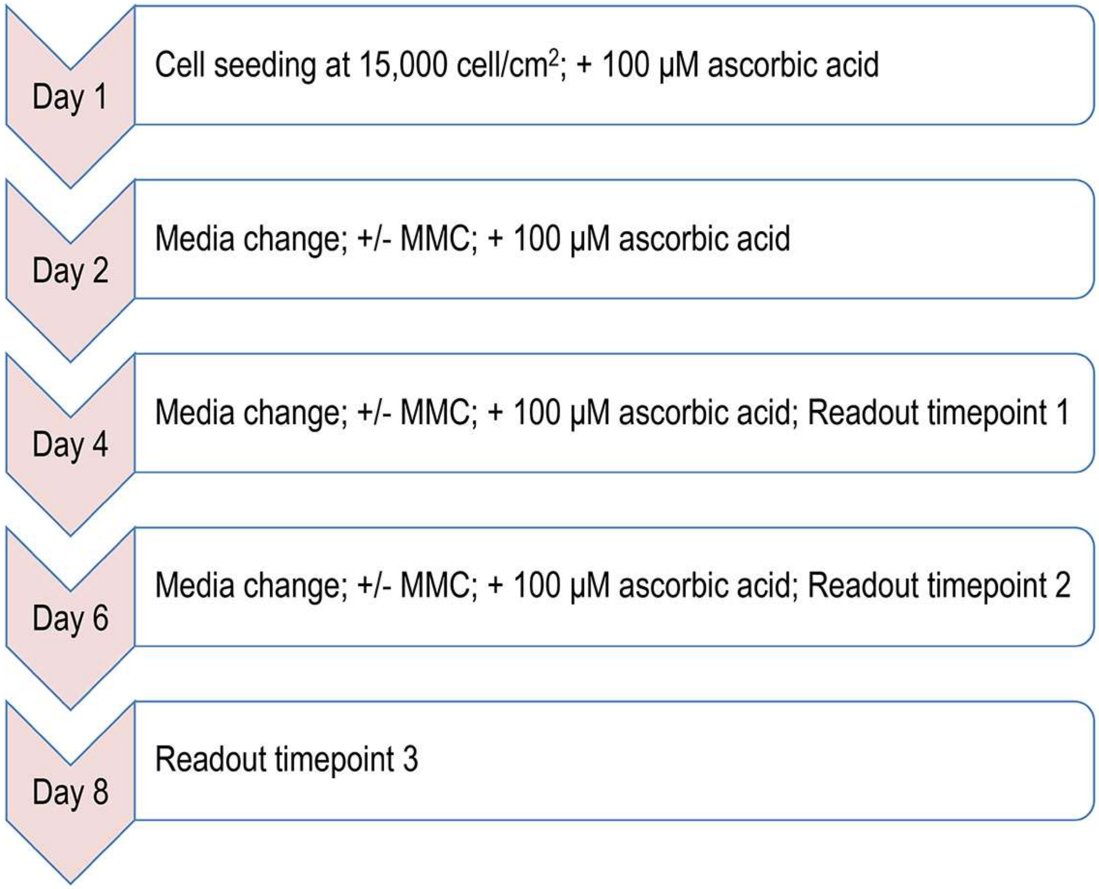

Figure S1. Experimental workflow of cell culture work and analysis.

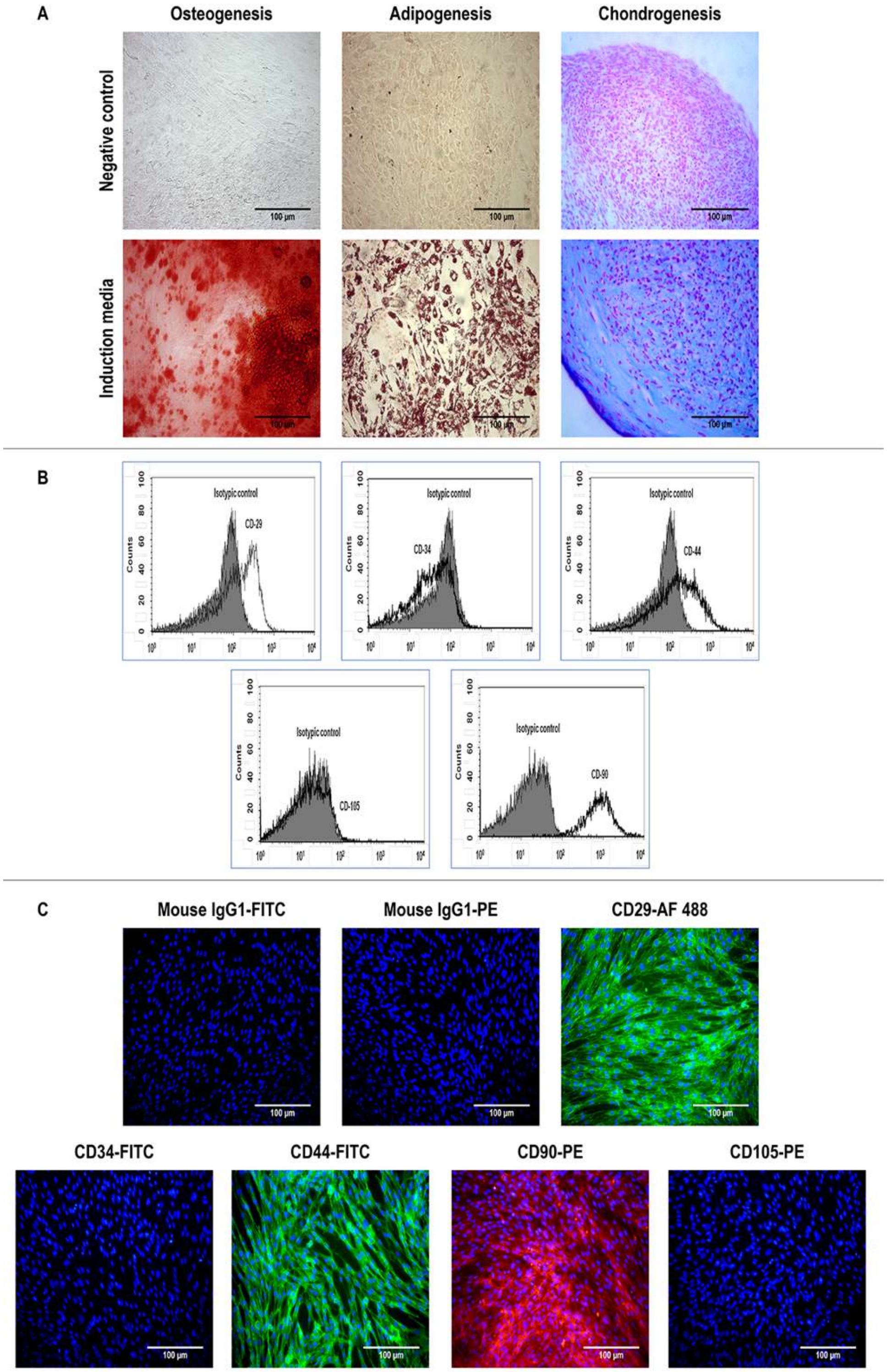

Figure S2. The extracted cells had the capacity to deposit calcium in osteogenic media, lipid droplets in adipogenic media and glycosaminoglycans in chondrogenic media (**A**). FACS analysis revealed that 95.2% ± 0.3% of the cells were positive for CD90, 58.4% ± 1.4% of the cells were positive for CD44 and 41.7% ± 11.5% of the cells were positive for CD29 and 0.1% ± 0.0% and 0.0% ± 0.0% of the cells were negative for CD105 and CD34, respectively (**B**). Immunocytochemistry analysis visually verified the FACS analysis data (**C**).

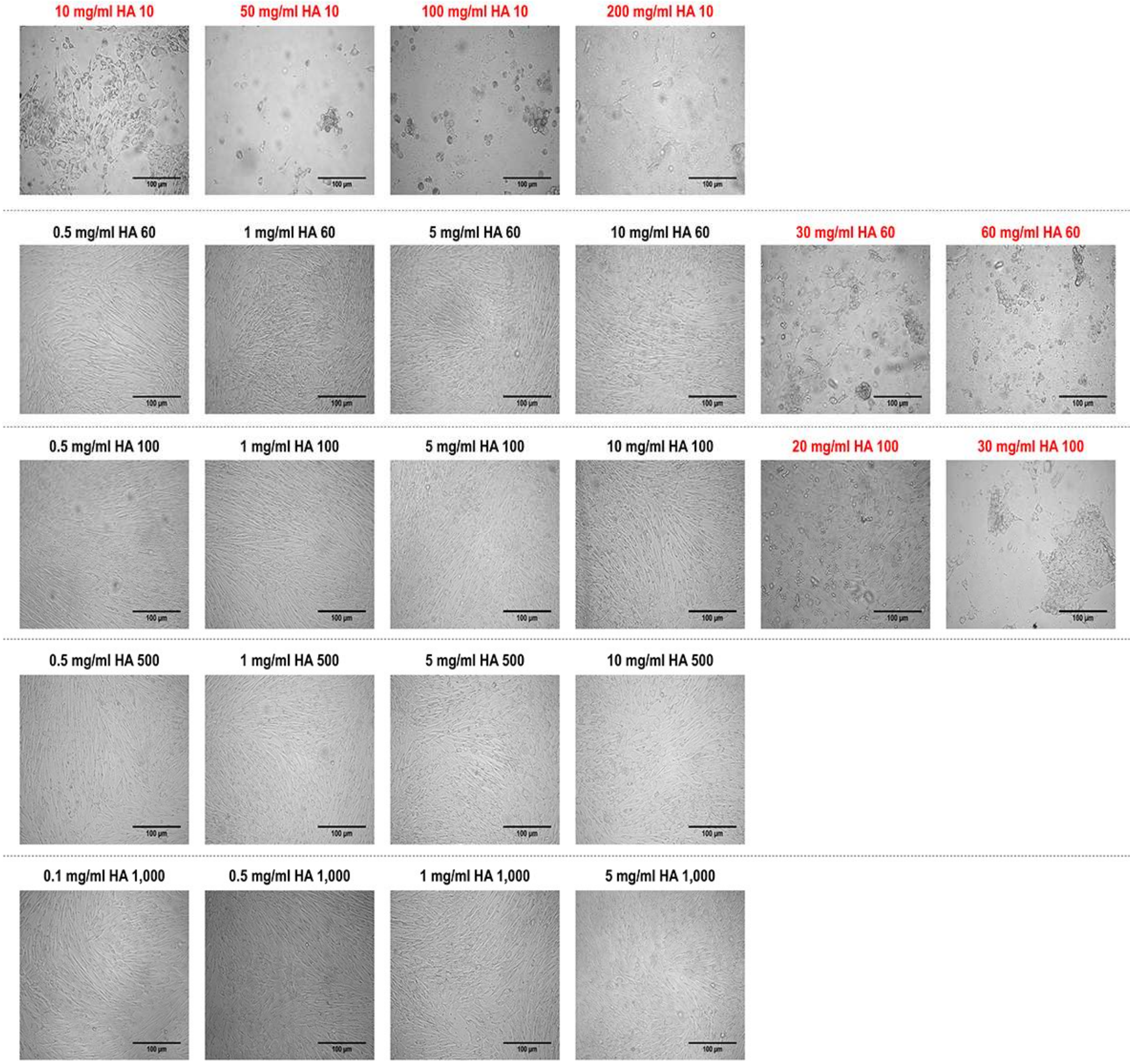

Figure S3. Bright field microscopy analysis after 4 days in culture revealed that up to 10 mg/mL concentrations of the HAs 60 kDa, 100 kDa and 500 kDa and up to 5 mg/mL concentrations of HA 1,000 kDa were completely soluble and allowed cell attachment and growth (black font). All concentration of HA 10 kDa and concentrations higher than 10 mg/mL of HA 60 kDa and 100 kDa (red font) resulted in cell detachment or microgel formation.

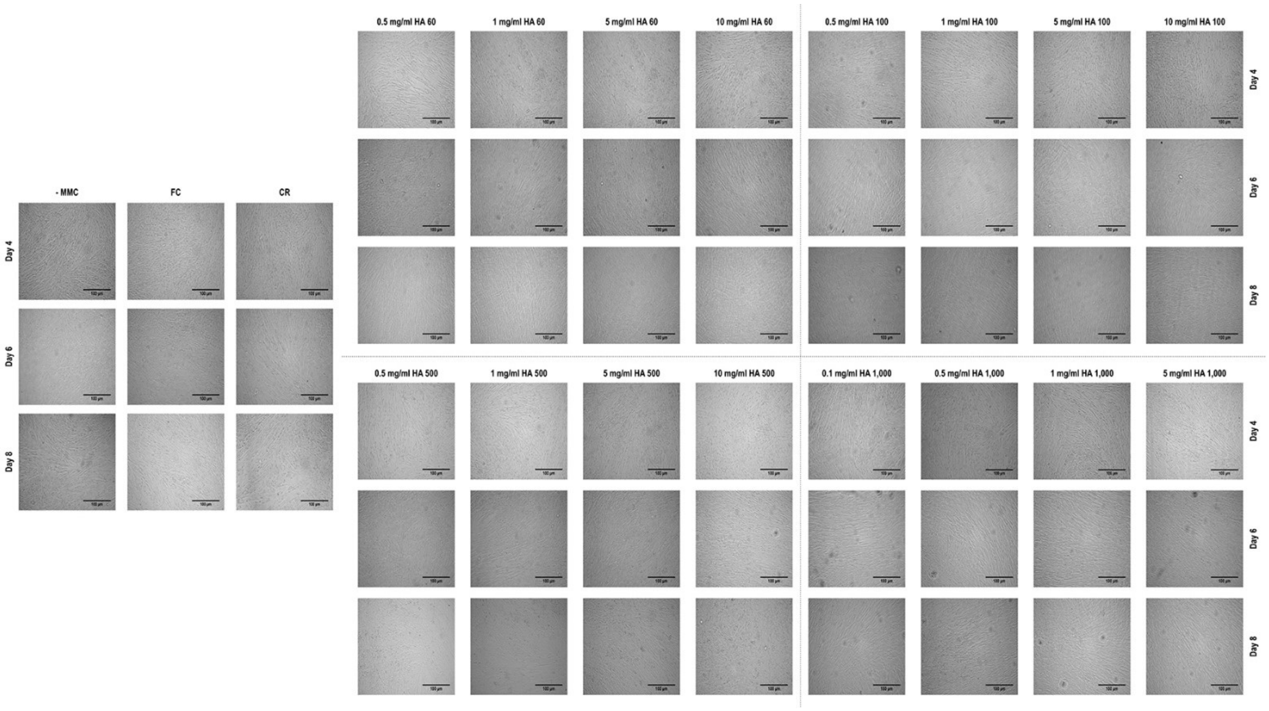

Figure S4. Bright field microscopy analysis revealed no apparent differences at any timepoint in cell morphology between the groups.

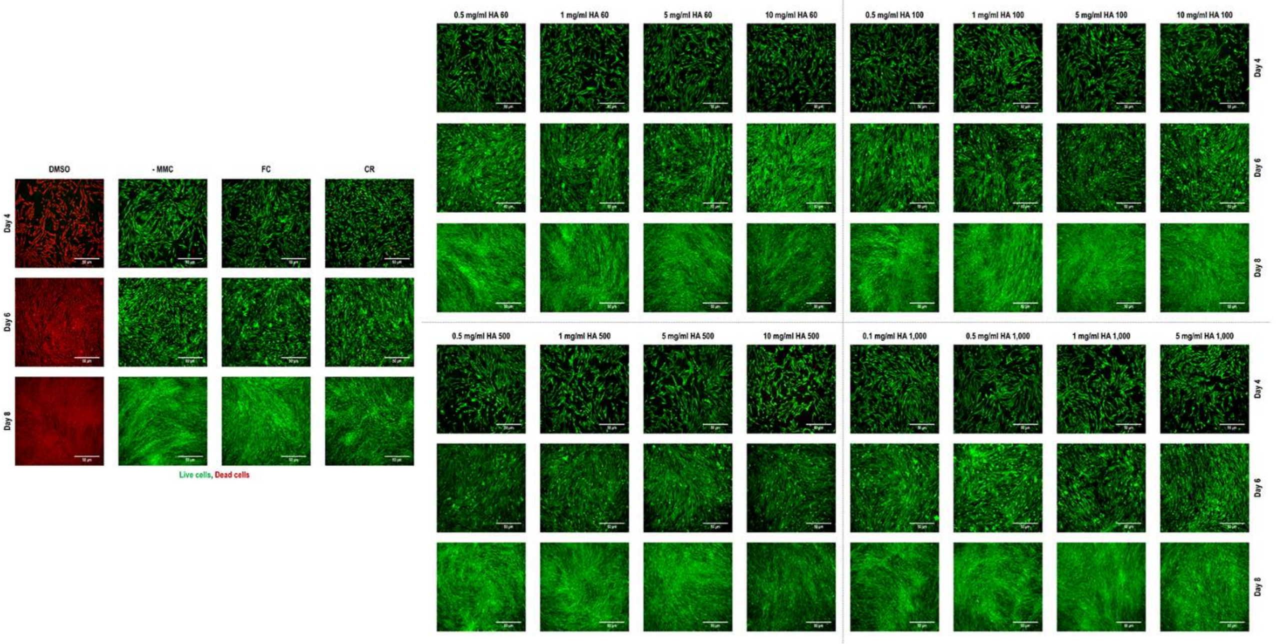

Figure S5. Qualitative cell viability analysis revealed no apparent differences at any timepoint in cell viability between the groups.

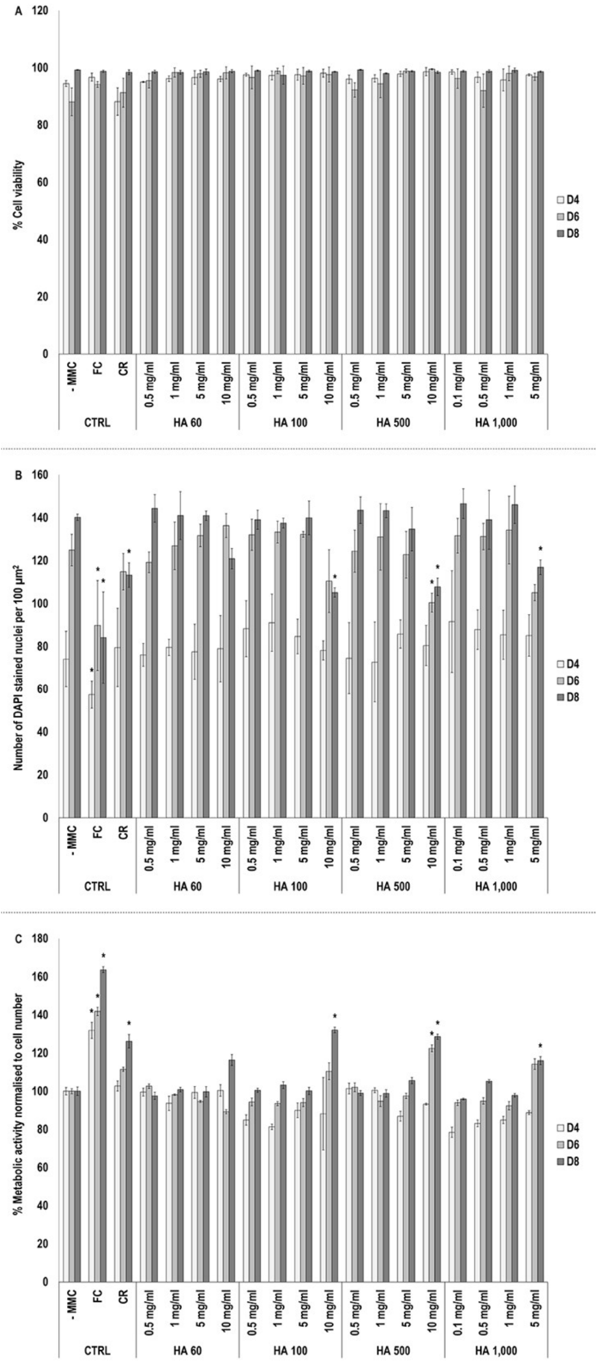

Figure S6. Quantitative cell viability analysis revealed no apparent differences at any timepoint in cell viability between the groups (**A**). Quantitative cell proliferation (**B**) and metabolic activity (**C**) analyses revealed that the Ficoll® at all timepoints significantly ($p < 0.05$) decreased cell number and increased cell metabolic activity. * indicates significant ($p < 0.05$) difference to the-MMC control group at a given timepoint.

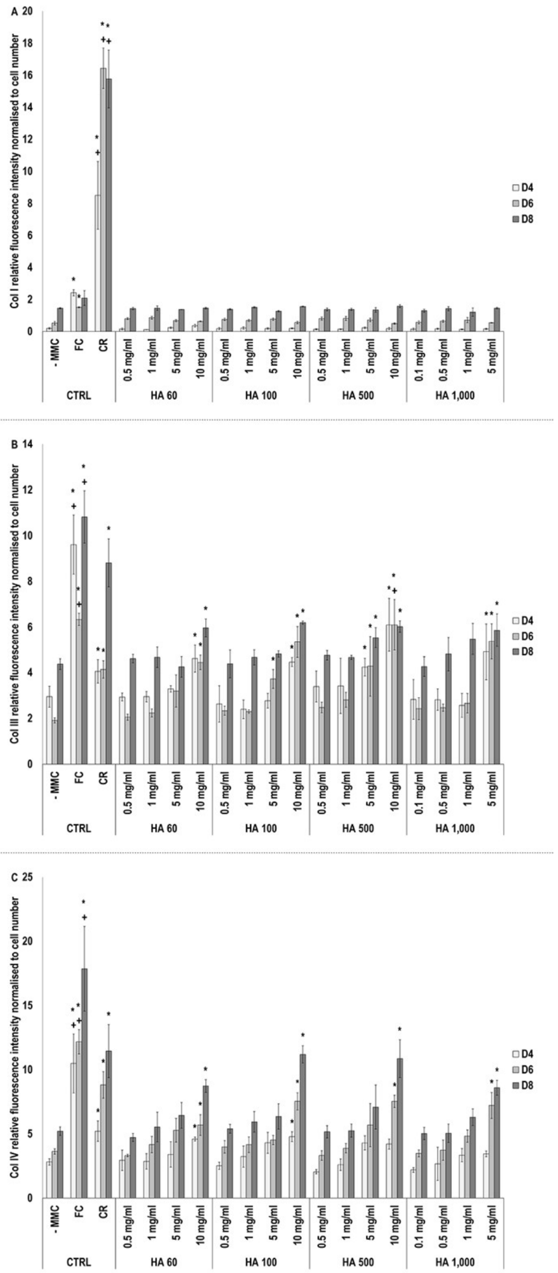

Figure S7. Fluorescence intensity analyses revealed that carrageenan induced the highest ($p < 0.001$) collagen type I deposition (**A**), Ficoll® induced the highest ($p < 0.001$) collagen type III deposition [at day 6, the 10 mg/mL HA 500 kDa group was not significantly ($p > 0.05$) different to the Ficoll® group] (**B**) and Ficoll® induced the highest ($p < 0.001$) collagen type IV deposition (**C**) than the non MMC group at all timepoints. * indicates significantly ($p < 0.05$) higher to the-MMC control group at a given timepoint. + indicates significantly ($p < 0.05$) higher to all groups at a given timepoint.
